# Diagnostic and prognostic role of NLR in testicular cancer

**DOI:** 10.37349/etat.2024.00270

**Published:** 2024-09-19

**Authors:** Shirin Sarejloo, Saghar Babadi, Shokoufeh Khanzadeh, Amirhossein Salimi, Alec Clark, Dinyar Khazaeli, Monireh Khanzadeh, Arshin Ghaedi, Brandon Lucke-Wold

**Affiliations:** Norte University, Paraguay; IRCCS Istituto Romagnolo per lo Studio dei Tumori (IRST) “Dino Amadori”, Italy; ^1^Cardiovascular Research Center, Shiraz University of Medical Sciences, Shiraz 767545, Iran; ^2^Student Research Committee, Ahvaz Jundishapur University of Medical Science, Ahvaz 868765, Iran; ^3^Student Research Committee, Tabriz University of Medical Sciences, Tabriz 585658, Iran; ^4^Student Research Committee, Shahid Sadoughi University of Medical Sciences, Yazd 876576, Iran; ^5^University of Central Florida College of Medicine, Orlando, FL 32608, USA; ^6^Urology Department, Imam Khomeini Hospital, Ahvaz 687667, Iran; ^7^Geriatric & Gerontology Department, Medical School, Tehran University of Medical and Health Sciences, Tehran 865476, Iran; ^8^Student Research Committee, School of Medicine, Shiraz University of Medical Sciences, Shiraz 767545, Iran; ^9^Department of Neurosurgery, University of Florida, Gainesville, FL 32608, USA

**Keywords:** Neutrophil to lymphocyte ratio, testicular cancer, systematic review

## Abstract

**Background:**

To summarize the results of available studies for investigating the role of neutrophil to lymphocyte ratio (NLR) in testicular cancer (tCa).

**Methods:**

The search was conducted on PubMed, Scopus, and Web of Science up to November 21, 2021. Finally, a total of 31 studies were included in this review.

**Results:**

NLR was higher in tCa patients compared to healthy controls and benign testis pathologies, and decreased significantly after orchiectomy. An elevated NLR predicts poor prognosis, advanced stage, presence of nodal or distant metastases, contralateral tumor development, lower time-to-cancer specific death, worse OS, and poorer response to chemotherapy. However, NLR could not differentiate between seminomas and non-seminomatous tCa.

**Discussion:**

NLR has a significant diagnostic and prognostic value in tCa.

## Introduction

Testicular cancer (tCa) is the most prevalent cancer in men aged 20 to 40. It has a prevalence of 5.7 per 100,000 persons in developed countries [1–3]. Testicular germ cell tumor (TGCT) account for 90–95% of tCa [3]. There are six biological capacities acquired during the multi-stage growth of tumors, which is characteristic of cancer [4]. These include metastasis activation and invasion, induction of angiogenesis, replicative immortality, resistance to cell death, avoidance of growth suppressors, and maintenance of proliferative signaling [4]. Two more potential factors have been added to this list in the previous decade: avoiding immune destruction and reprogramming energy metabolism [4, 5]. It is believed that immune system dysfunction is linked to tumor microenvironmental inflammation that ultimately contributes to acquiring the tumor characteristics [5, 6]. Inflammation is one of the indications of cancer as a result of this process [6–9]. Cancer suppresses the immune system not only at the tumor location, but also throughout the body. Several markers of systemic inflammation (such as fibrinogen, albumin, calcitonin, CRP, and others) are routinely evaluated in these patients [10–12]; however, markers that are reproducible, affordable, and readily accessible from complete blood count results have gained traction [13–18]. Single-parameter biomarkers such as hemoglobin, CRP, leukocytes, lymphocytes, platelets, monocytes, and leukocytes have been shown to have predictive value for various malignancies [13–15, 19]. In addition to single biomarkers, relative ratios such as lymphocyte to monocyte ratio (LMR), platelet to lymphocyte ratio (PLR), and neutrophil to lymphocyte ratio (NLR) have been investigated during the past decade [20–24]. These ratios represent the distinct tumor-preventive and tumor-inducing activities of various immune cells. As a result, they are assumed to be predictive of a valid and comprehensive inflammatory response linked to the development and progression of a malignant tumor. Among these ratios, NLR has been the most widely investigated biomarker [25]. NLR has now been suggested as a prognostic factor by the European Association of Urology (EAU) guidelines, but its clinical applicability in prognostic scores is limited due to a lack of data [1]. NLR has been shown to be diagnostic and prognostic in a variety of cancers; including gastric, colorectal, breast, hepatocellular, and ovarian cancers [25]. In the context of urological tumors, a recent umbrella review showed that NLR has a substantial predictive value for urothelial upper tract carcinoma, non-muscle-invasive bladder cancer, muscle-invasive bladder cancer, renal cell carcinoma, and prostate cancer [1]. However, to the best of our knowledge, there is no systematic review on the role of NLR in tCa.

Because the burden of tCa is increasing globally among young men, the need for better diagnostics is indicated. Recently, a significant increase in the number of studies reporting NLR as a potential biomarker in this cancer has been reported [2, 4, 26–54]. The need for a systemic evaluation on whether the evidence surrounding the prognostic and diagnostic role of NLR in this cancer was warranted. As a result, we conducted a systematic review to compile all published evidence related to the role of the NLR in tCa patients to assist clinicians in better understanding the pathogenesis, differential diagnosis, staging, and predicting survival and outcome of this cancer.

## Materials and methods

### Literature search strategy

This study aimed to summarize prior research on the role of NLR in tCa. In compliance with PRISMA-2009 guidelines, the search was conducted utilizing three main databases: PubMed, Scopus, and Web of Science. The most recent update to the search was on November 21, 2021. Additional studies were found using the Google Scholar database. All publications were imported into the EndNote online application after the search, and duplications were removed. Two separate authors assessed the remaining studies for inclusion based on criteria; the authors were uninformed of each other’s judgments. Scanning abstracts and full-text articles were done in initial screening. Following the initial screening, two independent authors reviewed the full-text papers for final inclusion. The authors were unaware of each other’s thoughts, and any conflicts were finally resolved by a third reviewer. Table 1 presents the search strategy.

**Table 1:** Presents the search strategy

**Database**	**Keywords**	**Number of articles**
PubMed	((“neutrophil”[All Fields] AND “lymphocyte”[All Fields] AND “ratio”[All Fields]) OR “neutrophil-to-lymphocyte”[All Fields] OR “NLR”[All Fields]) AND “testicular”[All Fields] AND (“cancer”[All Fields] OR “tumor”[All Fields])	25
Scopus	((ALL ((neutrophil AND lymphocyte AND ratio) OR (neutrophil-to-lymphocyte) OR NLR))) AND ((TITLE-ABS-KEY (cancer)) OR (TITLE-ABS-KEY (tumor))) AND (TITLE-ABS-KEY (testicular))	52
Web of Science	All = ((neutrophil AND lymphocyte AND ratio) OR (neutrophil-to-lymphocyte) OR NLR) AND All = (cancer OR tumor) AND ALL = (testicular)	41

### Study selection

#### Inclusion criteria

1. Peer-reviewed original studies.
2. Studies including tCa patients.
3. Studies examining the role of NLR in differential diagnosis, staging, and predicting survival of this disease.

#### Exclusion criteria

1. Animal studies, letters to editors, reviews, case reports, and case series.
2. Studies with overlapping data.
3. Studies on derived NLR (the ratio of neutrophil count to white cell count—neutrophil count) from concurrent investigation of other inflammatory biomarkers, such as PLR.

### Data extraction

Two authors independently retrieved data from the articles, and when conflicts arose, a third person was consulted. From the article, the first author’s name, country, year of publication, sample size, study design, and cancer histology were all extracted. We also obtained data on the NLR value, including the mean, standard deviation (SD), hazard ratio (HR), correlation coefficient, odds ratio (OR), and the results of receiver operating characteristic (ROC) and Kaplan-Meier survival analysis. The NLR level was presented as mean ± SD or median [interquartile range (IQR)]. Because of their heterogeneity, these papers were not suitable for a meta-analysis. As a result, we adopted an integrated approach to our research.

### Statistical analysis

We used standardized mean difference (SMD) to conduct two meta-analyses:

1. Comparing the NLR level between patients with stage I tCa with those with stage II/III tCa.
2. Comparing the NLR level between tCa patients with and without metastasis.

A random-effect model was used due to high level of heterogeneity. We used STATA software [Stata 12 (Stata Corp, College Station, TX)] to conduct the analysis.

## Results

### Literature search and selection

**Figure 1:**
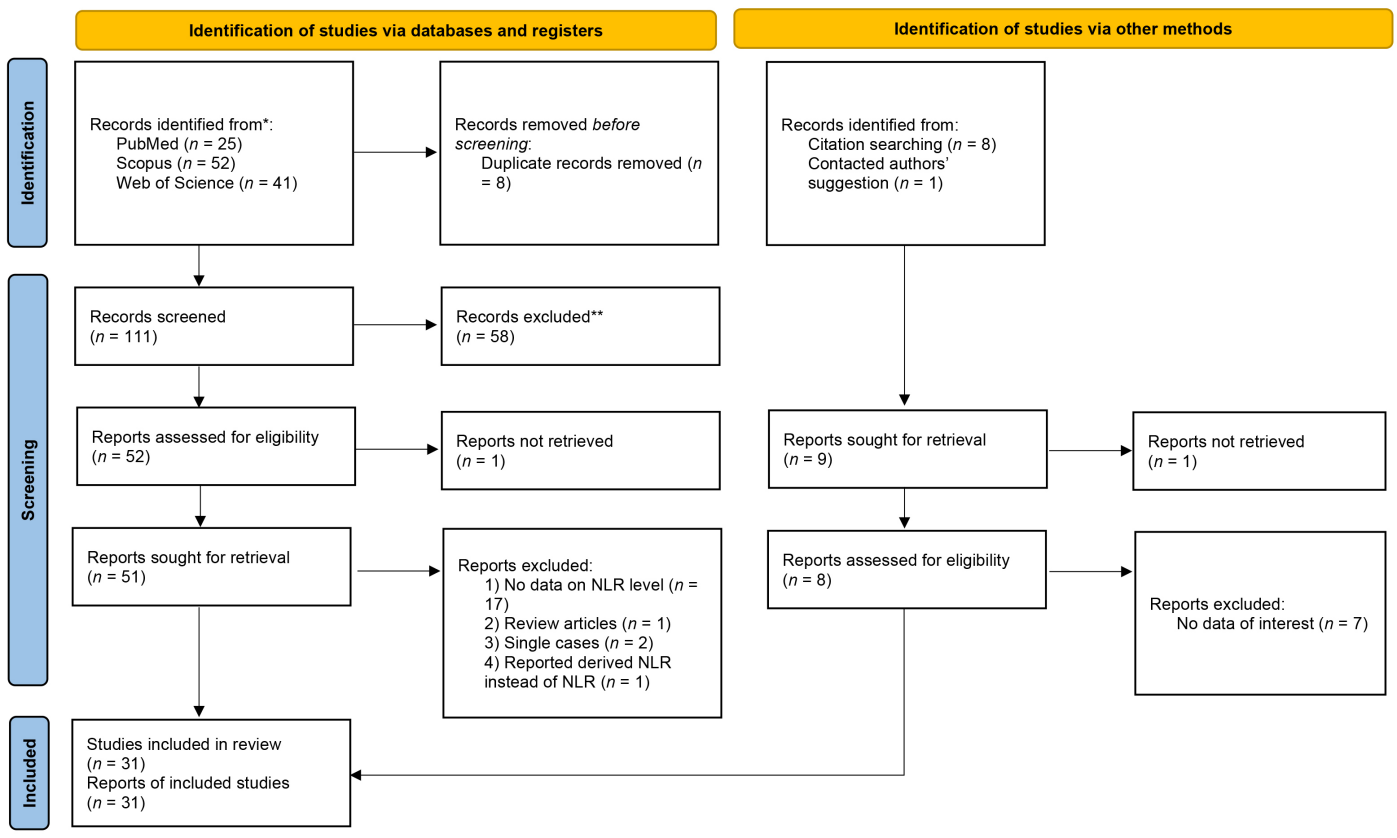
Flow chart of search and study selection

Figure 1 demonstrates the process of identifying and selecting papers in our study. The initial search yielded 25 PubMed records, 41 Web of Science records, and 52 Scopus records. The acquisition of 9 studies was obtained through other sources. Following the elimination of duplicate articles and an examination of the titles and abstracts of the remaining 111 articles, 53 articles were chosen for full-text analysis.

Due to a lack of data on NLR (*n* = 18), reporting derived NLR instead of NLR (*n* = 1), single case (*n* = 2), and review reports (*n* = 1), 22 of the 53 studies were removed after reading the entire text. As a result, a total of 31 studies [2, 4, 26–54] were included in the review.

### Characteristics of the included studies

There were 31 studies in total [2, 4, 26–54], all of which were in English and were retrospective in nature. Eleven studies evaluated the role of NLR in tCa diagnosis by comparing the NLR value of tCa patients with that of healthy individuals or patients with benign testis pathologies, such as varicocele, cryptorchidism, and atrophic testes [26–28, 30, 32, 33, 40, 42, 47, 49, 54]. Six studies reported the role of NLR in differentiating between different types of tCa [27, 36, 38, 42–44]. Furthermore, 12 studies showed the role of NLR in tCa staging [2, 4, 26, 33, 36, 38, 39, 41, 44, 45, 47, 52]. Thirteen studies on the association between NLR and nodal or distant metastases in tCa were found [2, 26, 28, 29, 36–38, 42–46, 51]. Thirteen studies investigated the role of NLR in predicting survival [cancer-specific survival (CSS), overall survival (OS), progression-free survival (PFS)], recurrence, and response to treatment in tCa [2, 26, 27, 29, 31, 35, 39, 44–46, 48, 50, 53]. Six studies demonstrated the association between NLR and tumor size in tCa [2, 34, 37, 42–44]. The association between NLR and conventional tumor markers of tCa (AFP, B-HCG, and LDH) was evaluated in four studies. In addition, two studies investigated the dynamic change in NLR after orchiectomy [37, 46].

### NLR in tCa diagnosis

Eleven studies evaluated the role of NLR in tCa diagnosis by comparing the NLR value of tCa patients with that of healthy individuals or patients with benign testis pathologies such as varicocele, cryptorchidism, and atrophic testes [26–28, 30, 32, 33, 40, 42, 47, 49, 54] (Table 2).

**Table 2:** General characteristics of studies on the role of NLR in tCa diagnosis

**Reference**	**Sample size**	**Study design**	**Year of publication**	**Country**	**Histopathology**	**Cut off value of NLR**	**Seminomatous tCa**	**Outcome**
Arıman and Merder [27]	152	Retrospective cohort	2021	Turkey	tCa	2.39	52.60%	NLR in healthy control group patients was significantly lower than patients with tCa.
Arda et al. [26]	182	Retrospective cohort	2020	Turkey	TGCT	1.78	46.60%	B-HCG level was significantly higher in the patients with higher NLR.
Başer and Aras [28]	83	Retrospective cohort	2020	Turkey	TGCT	2.27	60.00%	NLR values of the tCa patients were significantly higher than healthy controls.
Çalışkan et al. [30]	285	Retrospective cohort	2017	Turkey	tCa	1.64	37.00%	The NLR level was significantly higher in tCa group than those with benign pathology.
Kopru et al. [42]	80	Retrospective cohort	2019	Turkey	tCa	-	51.51%	Patients with tCa had a higher NLR compared to the control group with benign pathology.
Selvi and Başar [49]	96	Retrospective cohort	2020	Turkey	tCa	-	50.00%	NLR was significantly higher in patients with tCa than those with benign testis masses.
Girgin et al. [32]	76	Retrospective cohort	2021	Turkey	tCa	2.06	34.21%	The NLR values in patients with tCa were significantly higher than those with varicocele.
Gokcen et al. [33]	121	Retrospective cohort	2018	Turkey	TGCT	2.25	-	NLR was significantly higher in patients with tCa than those with varicocele.
Kartal et al. [40]	160	Retrospective cohort	2020	Turkey	TGCT	2.25	43.58%	NLR was significantly higher in patients with tCa than in the varicocele patients.
Şahin et al. [47]	291	Cross-sectional retrospective	2019	Turkey	tCa	3.16	-	NLR was significantly higher in patients with testicular tumor than in those with varicocele.
Yuksel et al. [54]	72	Retrospective cohort	2016	Turkey	tCa	2.06	16.66%	NLR was statistically higher in patients with tCa compared with varicocele patients.

tCa: testicular cancer; TGCT: testicular germ cell tumor; NLR: neutrophil to lymphocyte ratio

In the study conducted by Arıman and Merder [27], the NLR level of 152 tCa patients and 100 healthy controls were compared. NLR in healthy control group patients was significantly lower than tCa patients (1.69 ± 0.51 and 2.69 ± 2.25, respectively, *P* < 0.001). In the ROC analysis, the cut-off for NLR in distinguishing between cancer patients and healthy controls was found to be 2.39 [sensitivity = 54.00%, specificity = 90.00%, area under the curve (AUC) = 0.763, *P* < 0.001].

Arda et al. [26] reported similar results (1.6 ± 1.05 and 2.37 ± 2.02, respectively, *P* < 0.001) in a study of 90 tCa patients and 92 healthy controls. The best cut-off point of NLR was 1.78 (sensitivity = 81.80%, specificity = 55.40%, AUC = 0.711, *P* < 0.001). These results matched those observed by Başer and Aras [28] on 40 tCa patients and 43 healthy controls (3.45 ± 3.19 vs. 1.73 ± 0.51, *P* = 0.001). The ROC curve was drawn for the NLR in tCa diagnosis. The best cut-off point of NLR was 2.27 (sensitivity = 55%, specificity = 88.5%, AUC = 0.687, *P* = 0.003).

In the study by Çalışkan et al. [30], the NLR level data of 126 tCa patients and 159 patients undergoing orchiectomy for atrophic testes and cryptorchidism without any inflammation and malignancy were compared. The NLR level was significantly higher in the tCa group than in the group undergoing orchiectomy for atrophic testes and cryptorchidism (4.54 ± 3.89 and 3.44 ± 2.89, respectively, *P* = 0.006). In ROC analysis, the best cut-off value for NLR was 1.64 with a sensitivity of 98.41%, specificity of 28.30%, and AUC of 0.645. They declared that the diagnostic value of NLR was higher than that of absolute lymphocyte count (*P* = 0.03) and was similar to that of absolute neutrophil count (*P* = 0.40).

Kopru et al. [42] performed an analysis of 142 patients undergoing orchiectomy in their center. Sixty-six patients were diagnosed with a malignant testicular tumor and 14 patients with benign orchiectomy pathology. Sixty patients were excluded because they were diagnosed with other malignancies. Patients with tCa had a higher NLR [median (IQR) = 2.35 (1.62, 3.65)] compared to the control group [median (IQR) = 1.55 (1.02, 2.01), *P* = 0.005].

In addition, Selvi and Başar [49] compared 20 patients with malignant masses and 11 patients with benign masses and found similar results [2.35 (1.55–4.19) vs. 1.67 (1.33–2.12), *P* = 0.036].

In the study by Girgin et al. [32], the data of 76 male patients (38 varicocele patients and 38 patients with localized tCa) were analyzed. The NLR values in patients with tCa were significantly higher than in the varicocele patients (mean ± SD = 3.53 ± 2.29 vs. 2.07 ± 0.73, *P* = 0.001). The AUC for the diagnostic role of NLR was 0.74 with a cut-off value of 2.06, 68% sensitivity, and 64% specificity.

This finding is in agreement with the findings of Gokcen et al. [33], which showed that NLR was significantly higher in tCa patients than the varicocele group (3.1 ± 1.4 and 2.0 ± 1.5 respectively, *P* ≤ 0.001). NLR cut-off of 2.25 with a sensitivity of 0.667%, specificity of 0.744% was accepted as a differentiating cut-off in testicular tumors.

These findings are further supported by a study by Kartal et al. [40] on 39 patients with localized TGCT and 121 control patients undergoing varicocelectomy that reported similar results [2.6 (2.0–3.6) vs. 1.8 (1.4–2.4), *P* ≤ 0.001]. In the ROC analysis performed for the NLR, the best cut-off of NLR in distinguishing the two groups was calculated to be 2.25 (sensitivity = 66.7%, selectivity = 73.6%, AUC = 0.71).

Also, Şahin et al. [47] reported similar results. They included 120 patients with testicular tumors and 171 control groups undergoing varicocelectomy. The NLR of the tumor group was significantly higher than the varicocele group (4.22 ± 3.54, 3.49 ± 2.79, *P* = 0.001). The cut-off point for NLR in the diagnosis of the testicular tumor was > 3.16. The sensitivity of this value was 63.87%, the specificity was 63.16%, and AUC was 0.612 (*P* = 0.001).

Furthermore, the findings of the Yuksel et al. [54] study, including 72 males (36 tCa, and as a control group 36 varicocele patients), mirror those of previous studies (3.18 ± 1.76 vs. 1.99 ± 1.17, *P* = 0.001). The area under the ROC curve for NLR in localized tCa patients was 0.74, with a threshold value of 2.06 and sensitivity = 69% and specificity = 69%.

#### Final comment

Based on the findings of the studies stated above, patients with cancer had a higher value of NLR compared to either healthy patients or those with benign pathologies. So, we may infer a strong link between systemic inflammation assessed by NLR and tCa. In addition, NLR appears to be an independent predictor of malignancy of testicular masses.

### NLR in differentiating between different types of tCa

Six studies reported the role of NLR in differentiating between different types of tCa [27, 36, 38, 42–44] (Table 3).

**Table 3:** General characteristics of studies on the role of NLR in differentiating between different types of tCa

**Reference**	**Sample size**	**Study design**	**Year of publication**	**Country**	**Histopathology**	**Cut off value of NLR**	**Seminomatous tCa**	**Outcome**
Arıman and Merder [27]	152	Retrospective cohort	2021	Turkey	tCa	2.39	52.60%	There was no statistically significant difference between cases with seminoma and non-seminomatous tCa.
Jankovich et al. [38]	103	Retrospective cohort	2017	Slovakia	TGCT	4	39.80%	There was no statistically significant difference in NLR level between patients with seminomas and non-seminomatous tCa.
Kopru et al. [42]	80	Retrospective cohort	2019	Turkey	tCa	-	51.51%	No statistically significant difference, was found in NLR of patients with seminomas and non-seminomatous tCa groups.
Köşeci et al. [43]	72	Retrospective cohort	2021	Turkey	TGCT	2.5	43.00%	NLR level wasn’t significantly different between seminomas and non-seminomatous tCa groups.
Olcucu et al. [44]	99	Retrospective cohort	2020	Turkey	TGCT	3.21	45.45%	There was no significant correlation between NLR and different types of tCa.
Horsanali et al. [36]	128	Retrospective cohort	2017	Turkey	TGCT	3.72	44.80%	There was statistically significant difference in NLR between different tumor histopathology.

tCa: testicular cancer; TGCT: testicular germ cell tumor; NLR: neutrophil to lymphocyte ratio

In the study conducted by Arıman and Merder [27] on 152 tCa patients, there was no statistically significant difference between cases with seminoma and non-seminomatous tCa [2.28 (1.92–2.91) and 3.00 (1.68–5.38), respectively, *P* > 0.05].

Jankovich et al. [38], in a study on 103 TGCT, reported consistent results showing that there was no statistically significant difference in NLR level between patients with seminomas and non-seminomatous tCa either in the group with NLR ≥ 4 (*P* = 0.6698) or in the group with NLR < 4 (*P* = 0.9115).

Kopru et al. [42], in the investigation of 66 tCa patients, reported results similar to those observed in the studies by Arıman and Merder [27] and Jankovich et al. [38] [2.14 (1.56, 3.20) vs. 2.60 (1.74, 3.98), *P* = 0.25].

The present findings seem to be consistent with other research in that the cancer histopathology is not different between those with NLR < 2.5 and those with NLR ≥ 2.5 (*P* = 0.06) and between patients with NLR < 3.219 and those with NLR ≥ 3.219 (*P* = 0.363), as seen in the studies by Köşeci et al. [43] and Olcucu et al. [44].

However, the findings of Horsanali et al. [36] do not support the results mentioned earlier. They performed a study on 128 tCa patients and revealed a statistically significant difference in NLR between different types of tumor histopathology. In the patients who have NLR < 3.72, the majority of patients had seminoma histopathology, and for NLR ≥ 3.72, the majority of patients had seminomas histopathology (*P* = 0.017).

#### Final comment

Based on the findings of these investigations, we may conclude that NLR could not differentiate between different types of tCa, specifically between seminomas and non-seminomatous tCa.

### NLR in tCa staging

There are three stages of tCa. In stage 1, cancer is only in the testicle. In stage 2 the cancer has spread to nearby lymph nodes, and stage 3 tCa means that it has spread to distant parts of the body (metastasis) [55]. Twelve studies showed the role of NLR in tCa staging [2, 4, 26, 33, 36, 38, 39, 41, 44, 45, 47, 52] (Table 4).

**Table 4:** General characteristics of studies on the role of NLR in tCa staging

**Reference**	**Sample size**	**Study design**	**Year of publication**	**Country**	**Histopathology**	**Cut off value of NLR**	**Seminomatous tCa**	**Outcome**
Gokcen et al. [33]	121	Retrospective cohort	2018	Turkey	TGCT	2.25	-	No significant difference was found between patients in different stages according to their NLR values.
Şahin et al. [47]	291	Cross-sectional retrospective	2019	Turkey	tCa	3.16	-	There was no association between NLR and different tumor stages.
Arda et al. [26]	182	Retrospective cohort	2020	Turkey	TGCT	1.78	46.60%	Tumor stage was not different between patients with different values of NLR.
Horsanali et al. [36]	128	Retrospective cohort	2017	Turkey	TGCT	3.72	44.80%	No significant correlation was found between NLR value and tumor stage.
Yıldırım et al. [52]	21	Retrospective cohort	2013	Turkey	tCa	-	47.61%	There was no significant correlation between NLR and tCa staging.
Kölükçü et al. [41]	17	Retrospective cohort	2017	Turkey	tCa	-	17.64%	The NLR value was not different between different tumor stages.
Imamoglu et al. [4]	112	Retrospective cohort	2019	Turkey	TGCT	3.21	58.92%	There was a significant difference in NLR between stage I and advanced stages.
Jankovich et al. [38]	103	Retrospective cohort	2017	Slovakia	TGCT	4	39.80%	There was a significant correlation between NLR and tumor stage.
Karakaya et al. [39]	40	Retrospective cohort	2021	Turkey	TGCT	-	22.50%	Statistically significant positive correlations were found between tumor stage and NLR.
Olcucu et al. [44]	99	Retrospective cohort	2020	Turkey	TGCT	3.21	45.45%	Patients with advanced-stage cancer had higher values of NLR in comparison with those in lower stages.
Pęksa et al. [45]	180	Retrospective cohort	2021	Poland	TGCT	3.56	53.90%	NLR was significantly higher in patients with stage II/III tumors compared to those with stage I.
Tan et al. [2]	160	Retrospective cohort	2019	Singapore	tCa	3	63.80%	Patients with advanced stages had higher values of NLR than those in lower stages.

tCa: testicular cancer; TGCT: testicular germ cell tumor; NLR: neutrophil to lymphocyte ratio

Gokcen et al. [33], in the study of 39 tCa patients, found no significant difference between patients in different stages according to their NLR values (NLR of 3.1 ± 1.3, 2.1 ± 0.7, 3.4 ± 1.6 for stages 1, 2, and 3, respectively, and *P* = 0.247). Similarly, in the study of 120 tCa patients performed by Şahin et al. [47], there were not any differences between the tumor stages in terms of NLR (3.83 ± 2.58 in T1, 4.62 ± 4.94 in T2, 5.78 ±1.87 in T3, *P* = 0.108).

The findings of the aforementioned studies are consistent with those of Arda et al. [26] and Horsanali et al. [36] who reported that the tumor stage was not different between patients with NLR < 1.78 and those with NLR ≥ 1.78 (*P* = 0.475) and between patients with NLR < 3.72 and those with NLR ≥ 3.72 (*P* = 0.110) in the investigation of 90 and 128 tCa patients, respectively. Consistent results were reported by the Yildirim et al. [52] study on 21 patients with tCa, divided into group 1 = stage I and group 2 = stage II/III. They found no statisticfally significant difference between both groups regarding NLR as a predictive factor (1.99 ± 0.799 vs. 2.74 ± 1.484, respectively, *P* = 0.270).

Kölükçü et al. [41] enrolled 17 patients with tCa in their study and evaluated the role of NLR in determining tumor stage. They divided patients into two groups: group 1 contained stage T1, and group 2, which contained stage T ≥ 2 diseases. The NLR between groups 1 and 2 was not different [2.72 ± 1.86 and 3.05 ± 1.91 (*P* = 0.723)].

However, some published studies reported different results. Imamoglu et al. [4] conducted a study in which 112 tCa patients divided into two groups of seminoma and non-seminomatous tCa were analyzed. Results demonstrated that in the seminomas group, the difference in NLR level between patients in stage 1 tCa and advanced stage tCa (stage II and III) was significant [2.37 (1.71–3.72) and 4.39 (2.88–5.63), respectively, *P* = 0.012]. The best cut-off value for distinguishing between stage I and stage II/III was 3.21 (sensitivity of 69% and specificity of 75%, AUC of 0.776, *P* = 0.012). However, in the non-seminomatous group, no significant difference between different stages was seen [3.00 (1.93–4.65) in stage I vs. 3.30 (1.57–3.95) in stage II and III, *P* = 0.857]. Jankovich et al. [38] divided 103 tCa patients into two groups (patients with NLR ≥ 4 and < 4) and analyzed the groups separately. In the group with NLR ≥ 4, patients with stage > T1 had higher mean NLR than those with stage = T1 (*P* = 0.0105); however, in the group with NLR < 4, the difference was not significant (*P* = 0.0956). In the study conducted by Karakaya et al. [39] on 40 patients with tCa, statistically significant positive correlations were found between tumor stage and NLR (*r* = 0.505, *P* = 0.001). Similarly, Olcucu et al. [44] performed a study on 99 patients with tCa and found that patients with advanced-stage cancer had higher values of NLR in comparison with those in lower stages [2.2 (1.66–3.07) for stage I, 2.5 (2.07–3.88) for stage II, and 3.37 (2.47–6.86) for stage III, *P* = 0.002].

Pęksa et al. [45] reported similar results in a study of 180 patients with TGCT showing that NLR was significantly higher in patients with stage II/III tumors compared to those with stage I tumors (*P* < 0.001). In ROC analysis, the best cut-off value of NLR for the prediction of the stage was 3.56 (AUC = 0.65).

This significant difference was also reported by the study of Tan et al. [2] on 160 tCa patients. They revealed that in both seminomas and non-seminomatous cancers, patients in stage II A/B had higher NLR values than the patients in stage I (*P* = 0.036, OR = 3.00, 95% CI = 1.12–8.40 and *P* = 0.034, OR = 8.69, 95% CI = 2.41–13.84, respectively). Moreover, in both seminomas and non-seminomatous cancers, patients with advanced stages (stage IIC/III) had higher values of NLR than those in lower stages [*P* = 0.032, 95% CI = 4.50 (1.18–12.86) and *P* < 0.01, 95% CI = 13.60 (6.62–19.58), respectively].

#### Meta-analysis of differences between patients with stage I tCa and those with stage II/III tCa in NLR level

**Figure 2:**
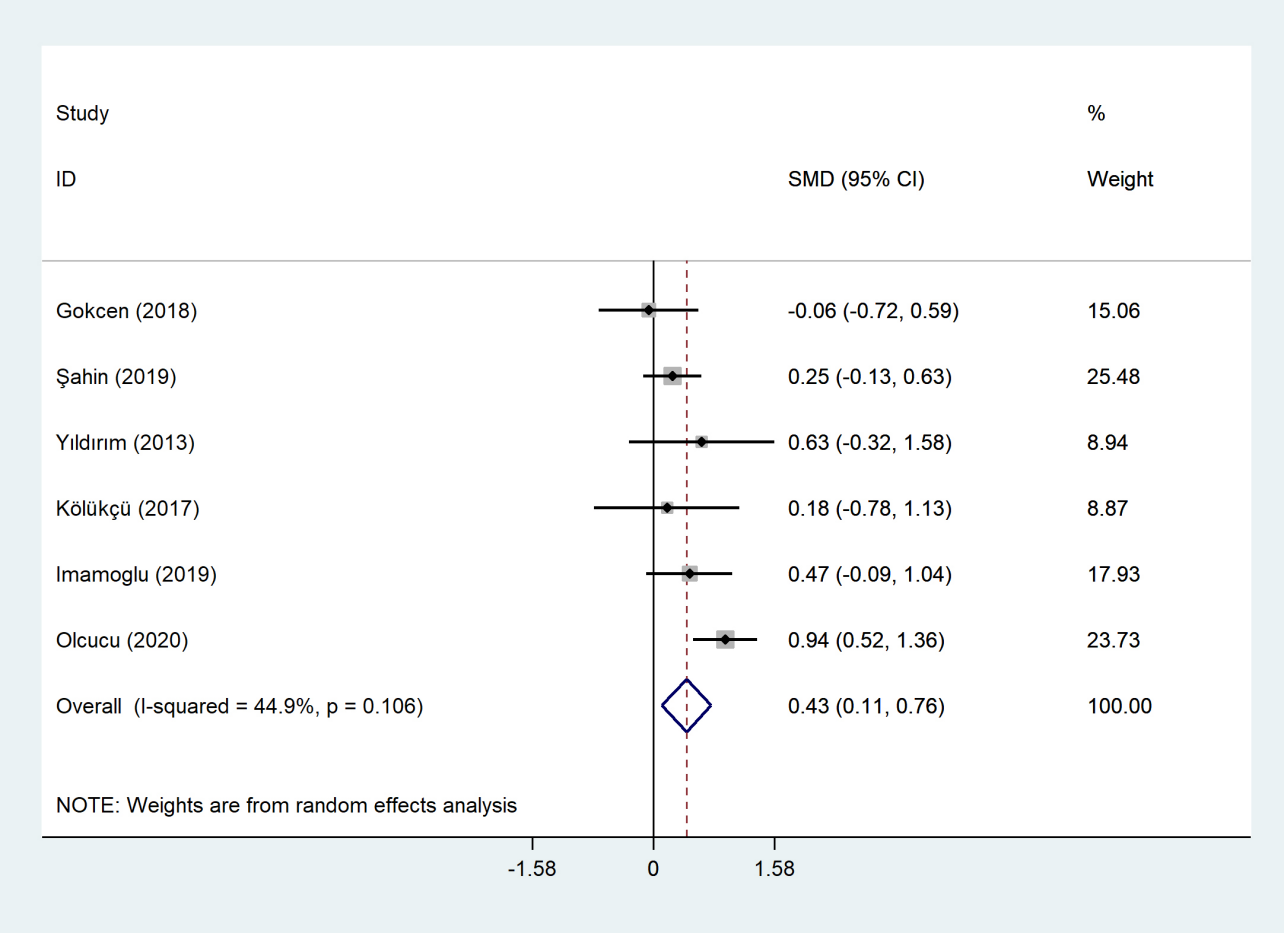
Meta-analysis of differences between patients with stage I tCa and those with stage II/III tCa in NLR level

Patients with stage II/III tCa had higher level of NLR compared to those with stage I tCa (SMD = 0.43, 95% CI = 0.11–0.76, *P* = 0.008, Figure 2).

#### Final comment

Based on the meta-analysis, NLR can predict tCa stage.

### NLR and nodal or distant metastases in tCa

Thirteen studies on the association between NLR and nodal or distant metastases in tCa were found [2, 26, 28, 29, 36–38, 42–46, 51] (Table 5).

**Table 5:** General characteristics of studies on the association of NLR with nodal or distant metastases in tCa

**Reference**	**Sample size**	**Study design**	**Year of publication**	**Country**	**Histopathology**	**Cut off value of NLR**	**Seminomatous tCa**	**Outcome**
Yigit et al. [51]	146	Retrospective cohort	2018	Turkey	tCa	3.11	36.30%	High level of NLR was associated with the risk of metastasis.
Bolat et al. [29]	53	Retrospective cohort	2017	Turkey	TGCT	3.35	50.90%	Elevated NLR could predict lymph node involvement.
Ilktac et al. [37]	61	Retrospective cohort	2020	Turkey	TGCT	2.56	59.00%	NLR was significantly higher in patients with non-localized TGCT compared to those with localized TGCT.
Horsanali et al. [36]	128	Retrospective cohort	2017	Turkey	TGCT	3.72	44.80%	Elevated NLR was significantly related to metastasis.
Köşeci et al. [43]	72	Retrospective cohort	2021	Turkey	TGCT	2.5	43.00%	Elevated NLR could predict metastasis.
Pęksa et al. [45]	180	Retrospective cohort	2021	Poland	TGCT	3.56	53.90%	Elevated NLR could predict the presence of nodal or distant metastases.
Bauzá Quetglas et al. [46]	80	Retrospective cohort	2020	Spain	tCa	2.25	62.50%	NLR had higher levels in patient with non-localized tCa than those with localized tCa.
Olcucu et al. [44]	99	Retrospective cohort	2020	Turkey	TGCT	3.21	45.45%	Elevated NLR could predict metastasis and low mean survival time.
Tan et al. [2]	160	Retrospective cohort	2019	Singapore	tCa	3	63.80%	High levels of NLR were associated with metastasis and poorer CSS.
Arda et al. [26]	182	Retrospective cohort	2020	Turkey	TGCT	1.78	46.60%	There was no significant association between metastasis with NLR of patients.
Jankovich et al. [38]	103	Retrospective cohort	2017	Slovakia	TGCT	4	39.80%	No significant correlation was found between metastasis with NLR.
Kopru et al. [42]	80	Retrospective cohort	2019	Turkey	tCa	-	51.51%	NLR had no statistically significant correlation with metastasis and the presence of lymphovascular invasion.
Başer and Aras [28]	83	Retrospective cohort	-	Turkey	TGCT	2.27	60.00%	There was no significant association between NLR and metastasis or lymph node involvement.

tCa: testicular cancer; TGCT: testicular germ cell tumor; NLR: neutrophil to lymphocyte ratio

In the study conducted by Yigit et al. [51], 108 patients with non-metastatic tCa and 38 patients with lymph node and solid organ metastasis were compared in terms of NLR level. There was a statistically significant difference in NLR level between non-metastatic and metastatic patients [3.3 (1.04–21.9) and 4.9 (1.5–14.5) respectively, *P* < 0.003]. They concluded that pre-operative NLR was helpful to predict lymph node and solid organ metastasis in patients with tCa. In the ROC analysis, the best cut-off point for the prediction of metastasis was 3.11, with an AUC of 0.69 (*P* < 0.001). Similarly, in the study by Bolat et al. [29] on 53 patients with tCa, NLR ≥ 3.55 was related to lymph node involvement at the time of diagnosis (*P* = 0.045). These results are also in accordance with the observations of Ilktac et al. [37] on 61 patients with TGCT, which showed that pre-operative and post-operative NLR was significantly higher in patients with non-localized TGCT compared to those with localized TGCT (pre-operative NLR of 3.83 ± 1.65 and 2.78 ± 1.84, respectively (*P* = 0.016), post-operative NLR of 1.57 ± 0.58 and 3.52 ± 2.79, respectively (*P* = 0.004)). In the ROC analysis for the presence of non-localized TGCT, the best cut-off point for pre-operative NLR was 2.56 (sensitivity = 75%, specificity = 60%, and AUC = 0.703). ROC analysis for post-operative NLR was not reported. In addition, the study by Horsanali et al. [36] on 128 tCa patients reported matching results. They assessed lymphovascular invasion, rete testes invasion, spermatic cord invasion, epididymal invasion, lymph node invasion, and distant metastasis in patients with NLR < 3.72 and NLR ≥ 3.72. The rates of rete testes invasion, lymph node invasion, and distant metastasis were higher in patients with NLR ≥ 3.72 (*P* = 0.004, < 0.001, and < 0.001, respectively). In addition, in the group with NLR < 3.72, 74.70% of patients were N0 (without lymph node invasion), 8.00% were N1, 5.70% were N2 and 11.50% were N3. For those in the group with NLR ≥ 3.72 N0 34.10%, N1 12.20%, N2 17.10% and N3 36.60% (*P* < 0.001). Further support on the association between NLR and metastasis was provided by Köşeci et al. [43] and Pęksa et al. [45]. Köşeci et al. [43] divided 72 tCa patients into two groups based on the presence of lymph node invasion and subsequently compared them in terms of NLR level. NLR was significantly lower in patients without lymph node invasion compared to those with lymph node invasion (2.25 ± 1.06 and 3.19 ± 2.32, respectively, *P* = 0.04). In the ROC analysis, the NLR value of 2.5 was determined as the cut-off value to assess lymph node status. For the patients with an NLR ≥ 2.5, lymph node invasion was significantly higher than in patients with NLR < 2.5 (68% and 31.9%, *P* = 0.003). Further, they divided patients with lymph node invasion into three groups: N1 (cancer has spread to at least one nearby lymph node with a size of < 2 cm), N2 (cancer has spread to at least one nearby lymph node with a size of 2 cm to 5 cm) and N3 (cancer has spread to at least one nearby lymph node with a size of > 5 cm). When they used 2.5 as a cut-off of NLR to compare these groups, N3 disease was significantly higher in patients with NLR ≥ 2.5 compared with the patients with NLR < 2.5 (28% and 6.4% respectively, *P* = 0.005). Pęksa et al. [45] analyzed the data of 180 patients with TGCT. They found that higher NLR was associated with the presence of nodal or distant metastases, in accordance with previous studies. In addition, in the study by Bauzá Quetglas et al. [46], 80 patients with tCa were included and divided into patients with localized disease and those with disseminated disease. Patients with localized disease had lower levels of NLR either before or after orchiectomy compared with those with non-localized disease [pre-orchiectomy NLR = 1.94 (1.35–2.56) and 3.33 (0.39–4.53), respectively, *P* = 0.001, post-orchiectomy NLR =1.64 (1.26–2.24) and 2.4 (1.49–3.44), respectively, *P* = 0.021]. Olcucu et al. [44] enrolled 99 tCa patients in their study and observed similar findings. Patients with metastasis had higher NLR than those without metastasis [3.38 (2.48–6.87) and 2.37 (1.73–3.43), respectively, *P* = 0.002]. In addition, those with retroperitoneal lymph node invasion had a higher level of this marker than those without retroperitoneal lymph node invasion [2.83 (2.27–4.93) and 2.22 (1.66–3.13) respectively, *P* = 0.004]. When dividing patients into two groups based on NLR level, there was a significantly higher rate of retroperitoneal lymph node invasion and metastatic disease in the group with NLR ≥ 3.219 (*P* = 0.015 and 0.005, respectively). Finally, Tan et al. [2] reviewed the data of 160 patients with tCa, which investigated the association of NLR and extra-testicular disease. The NLR value of 3.0 was considered the best cut-off point for evaluating extra-testicular disease, with a sensitivity of 74.4%, specificity of 77.8%, and AUC of 0.755. In multivariate analyses, NLR ≥ 3.0 was associated with lymph node invasion in patients with either pure seminomas or non-seminomatous TGCT (*P* = 0.031; OR = 2.91; 95% CI = 1.67–5.83 and *P* = 0.038; OR = 4.12; 95% CI = 1.26–6.51, respectively). In addition, NLR ≥ 3.0 was associated with metastasis in these patients (*P* = 0.041; OR = 2.48; 95% CI = 1.22–3.98, and *P* = 0.043; OR = 2.21; 95% CI =1.17–3.65, respectively).

Meanwhile, some previous studies were unable to demonstrate the link between NLR and metastasis. In the study conducted by Arda et al. [26], a total of 90 patients with tCa were included. Metastasis rate in patients with NLR < 1.78 was similar to those with NLR > 1.78 (8.00% and 13.85%, *P* = 0.721).

Jankovich et al. [38] divided 103 tCa patients into two groups: patients with NLR ≥ 4 and < 4 and analyzed the groups separately. In the group with NLR < 4, patients with metastasis had a higher mean NLR than those without metastasis (*P* < 0.001); however, in the group with NLR ≥ 4, the difference was not significant (*P* = 0.2008). Kopru et al. [42] performed a study on 66 tCa patients and observed that NLR had no statistically significant correlation with rete testis invasion and the presence of lymphovascular invasion.

In addition, in the study by Başer and Aras [28], a total of 40 tCa patients were included to determine whether there is a correlation between NLR and the prognostic risk factors previously identified for occult metastatic disease in tCa (tumor size, invasion of the rete testis, lymphovascular invasion in peri-tumoral tissue, proliferation rate > 70%, embryonal carcinoma percentage > 50%). Among these risk factors, only tumor size was correlated with NLR (correlation coefficient = 0.556, *P* < 0.001).

#### Meta-analysis of differences between tCa patients with and without metastasis

**Figure 3:**
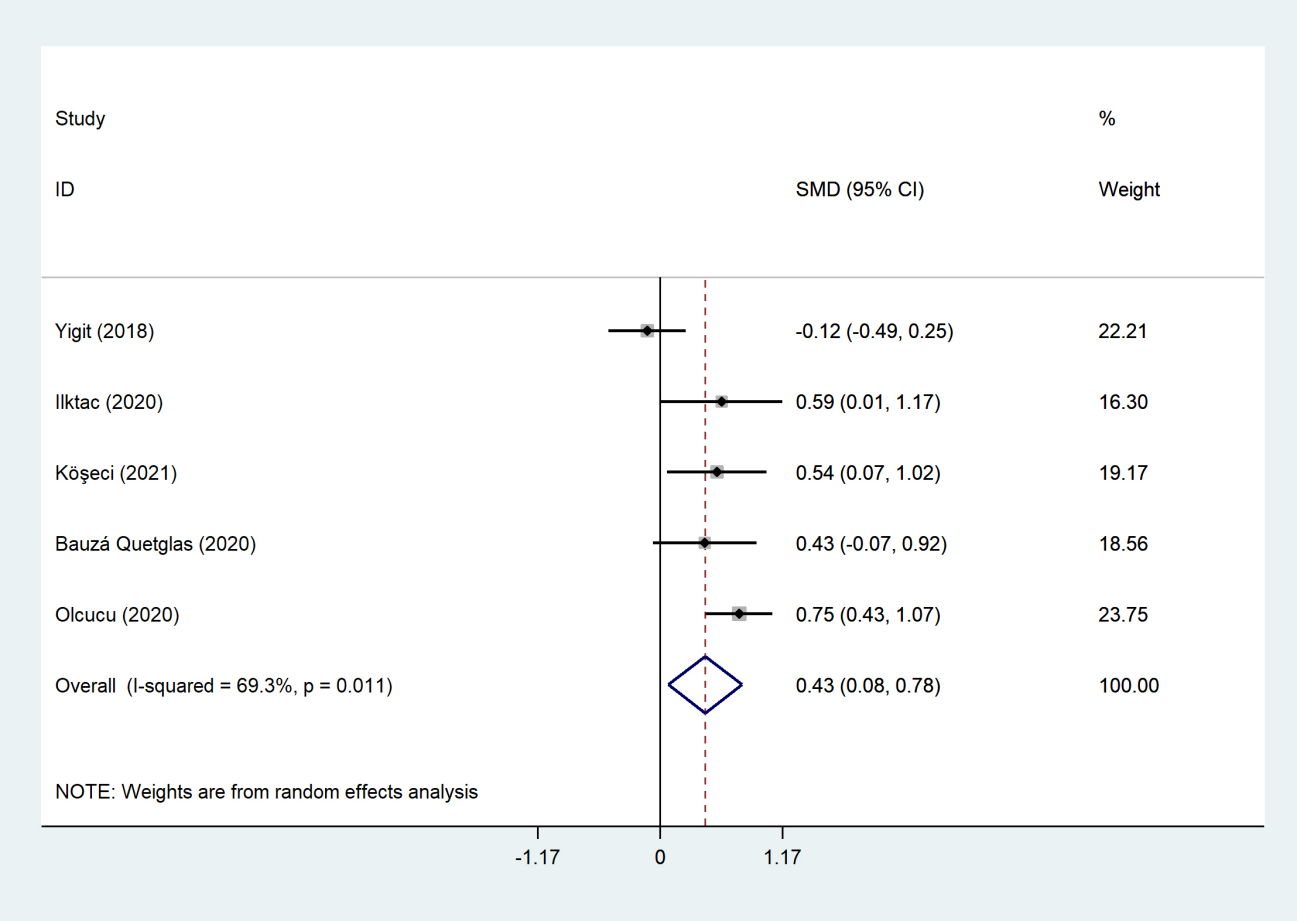
Meta-analysis of differences between tCa patients with and without metastasis

Patients with metastatic tCa had significantly higher level of NLR compared to those without metastasis (SMD = 0.43, 95% CI = 0.08–0.78, *P* = 0.016, Figure 3).

#### Final comment

Based on the review, the level of NLR in tCa patients can be associated with the presence of nodal or distant metastases. Studies have shown the NLR cut-off value ranging between 2.5 to 4 could predict the presence of metastases. A mediating mechanism by which high NLR contributes to tumor propagation and metastasis is through chronic inflammation. Neutrophils are dominant players in innate immunity that serve to amplify pro-inflammatory responses [56], whereas lymphocytes are components of the adaptive immune system which serve to regulate immune responses [57]. In the setting of a high NLR, the pro-inflammatory activity of neutrophils may outweigh the regulatory function of lymphocytes, allowing for unregulated peripheral inflammation to transmit onto tumoral tissue. Neutrophils have also been reported to produce tumor growth promoting compounds such as vascular endothelial growth factor, and thus may contribute to stimulating the tumor microenvironment through the induction of angiogenesis [58].

### NLR and survival in tCa

Thirteen studies investigated the role of NLR in predicting survival (CSS, OS, PFS), recurrence, and response to treatment in tCa [2, 26, 27, 29, 31, 35, 39, 44–46, 48, 50, 53] (Table 6).

**Table 6:** General characteristics of studies on the association of NLR with survival in tCa

**Reference**	**Sample size**	**Study design**	**Year of publication**	**Country**	**Histo** **pathology**	**Cut off value of NLR**	**Seminomatous tCa**	**Outcome**
Arıman and Merder [27]	152	Retrospective cohort	2021	Turkey	tCa	2.39	52.60%	Patients with good prognosis had statistically significant lower NLR than those with intermediate and poor prognosis.
Tan et al. [2]	160	Retrospective cohort	2019	Singapore	tCa	3	63.80%	High levels of NLR were associated with poorer CSS.
Fankhauser et al. [31]	146	Retrospective cohort	2018	Switzerland	TGCT	4.5	25.34%	NLR was an independent predictor of OS.
Olcucu et al. [44]	99	Retrospective cohort	2020	Turkey	TGCT	3.21	45.45%	Elevated NLR could predict metastasis and low mean survival time.
Herraiz-Raya et al. [35]	164	Retrospective cohort	2019	Spain	TGCT	4	50.00%	There was significant correlation between NLR and residual disease after chemotherapy and mean survival time of tCa patients.
Yang et al. [50]	2008–2016	Retrospective cohort	2019	China	tCa	2.66	-	High NLR was significantly associated with an elevated risk for disease progression.
Karakaya et al. [39]	40	Retrospective cohort	2021	Turkey	TGCT	-	22.50%	NLR was a strong predictor of response to chemotherapy in tCa patients.
Bauzá Quetglas et al. [46]	80	Retrospective cohort	2020	Spain	tCa	2.25	62.50%	Pre-orchiectomy NLR was higher in patients who had adjuvant chemotherapy than those without chemotherapy. Post-orchiectomy NLR was related to the risk of recurrence and recurrence-free survival.
Selvi and Başar [49]	99	Retrospective cohort	2019	Turkey	tCa	3.23	60.00%	NLR was found to be significantly higher in patient with bilateral tCa than those with unilateral tCa. High NLR could predict contralateral tumor development.
Arda et al. [26]	182	Retrospective cohort	2020	Turkey	TGCT	1.78	46.60%	CSS was not different between patients with different NLR.
Yoshinaga et al. [53]	63	Retrospective cohort	2021	Japan	TGCT	4.1	27.00%	There was no significant association between PFS and OS and NLR.
Pęksa et al. [45]	180	Retrospective cohort	2021	Poland	TGCT	3.56	53.90%	NLR could not predict progression or relapse in tCa patients.
Bolat et al. [29]	53	Retrospective cohort	2017	Turkey	TGCT	3.35	50.90%	Time-to-cancer specific death between patients with different NLR was not significantly different.

tCa: testicular cancer; TGCT: testicular germ cell tumor; NLR: neutrophil to lymphocyte ratio; CSS: cancer-specific survival; OS: overall survival; PFS: progression-free survival

In the study by Arıman and Merder [27] on 152 patients with tCa, patients with good prognosis, defined by using prognostic-based staging system for metastatic germ cell cancer, had statistically significant lower NLR [2.23 (1.71–2.98)] than those with intermediate [3.88 (2.91–4.42)] and poor prognosis [3.41 (2.60–5.38)] (*P* = 0.036 and *P* = 0.05, respectively). In ROC curve analysis, the cut-off point for NLR between the good prognostic group and the intermediate/poor prognostic group was 2.53 (sensitivity = 77.27%, specificity = 61.54%, AUC 0.704, *P* < 0.003).

Tan et al. [2], in their multivariate analysis, found that NLR ≥ 3.0 was independently associated with a poorer CSS (HR = 5.11, 95% CI= 1.68–11.42, *P* = 0.042). They divided patients into two groups: seminomas and non-seminomatous tCa. In the non-seminomatous cancer groups, the 5-year CSS was nearly 100% in patients with NLR < 3.0, but dropped significantly to 76% in patients with NLR ≥ 3.0 (*P* = 0.037; HR = 6.20; 95% CI = 1.834–13.16). However, in the seminoma group, there were no differences between the two groups (*P* = 0.58, HR = 4.68; 95% CI = 0.85–7.21). Fankhauser et al. [31] retrieved the data of 146 patients with metastatic TGCT undergoing first-line chemotherapy. Multivariable analyses showed that NLR ≥ 4.5 (AUC of 0.811, *P* < 0.001) was an independent predictor of OS [HR per 10 increase = 84.5 (95% CI = 2.2–3193.4), *P* = 0.017].

Similarly, Olcucu et al. [44], in survival analysis of the data of 99 tCa patients, found a statistically significantly lower OS time in the group with NLR ≥ 3.219 compared to those with NLR < 3.219 (77.41 months and 96.18 months, respectively, *P* = 0.008). They also performed a ROC analysis of NLR in predicting mortality and progression of the disease. The best optimal cut off point of NLR in predicting mortality and progression of the disease was 3.219 (sensitivity = 66.7%, specificity = 73.6%, AUC = 0.705 for mortality and sensitivity = 50%, specificity = 74.0%, AUC = 0.625 for progression). Herraiz-Raya et al. [35] conducted a study on 164 patients with TGCT and reported similar results. They showed that the mean OS time (year) in patients with NLR ≥ 4 was significantly lower in comparison to those with NLR < 4 (14.1 and 16.0, respectively, *P* = 0.035). Also, these patients’ mean progression time (year) was 13.1 and 14.8, respectively; however, the *P*-value was reported as non-significant. The NLR cut-off point of 4 as a predictive factor for disease progression had a specificity of 83.90%, sensitivity of 31.60%, and likelihood ratio of 2.00 %. Those values for disease survival were 37.5%, 83.1%, and 2.2%, respectively. In addition, patients with NLR ≥ 4, compared with those with NLR < 4 in the pre-operative hemogram, had a higher number of tumors at stages II and III (58.6% vs. 18.8%, *P* < 0.0001) and higher percentages of residual disease (48.4% vs. 9.8%, *P* < 0.0001) after chemotherapy. In the study by Yang et al. [50], patients with testicular diffuse large B-cell lymphoma were enrolled. The cut-off value based on PFS for NLR was 2.49. In the multivariate analysis for PFS, high NLR was shown to be independently associated with an elevated risk for disease progression in patients with testicular diffuse large B-cell lymphoma (HR = 9.069; 95% CI = 2.367–34.746; *P* = 0.001). Also, the cut-off value for NLR based on OS was 2.66. Univariate analysis of NLR at this cut-off revealed a significant correlation between NLR and OS (HR = 11.186; 95% CI = 1.356–92.275, *P* = 0.025). However, multivariate analysis showed no association. Karakaya et al. [39] screened the data of 40 tCa patients and evaluated the association between NLR and response to chemotherapy. In comparison to the complete response group, the mean NLR values were significantly higher in the other group (2.27 ± 1.44 vs. 3.48 ± 1.69, respectively, *P* = 0.02). In the study by Bauzá Quetglas et al. [46], 80 patients with tCa undergoing orchiectomy were included, and pre-orchiectomy and post-orchiectomy NLR were recorded. Pre-orchiectomy NLR was not associated with recurrence of tCa. However, post-orchiectomy NLR was higher in patients who had disease recurrence [2.51 (1.84–3.74) vs. 1.59 (1.10–2.24), *P* = 0.001]. A stage disease-stratified analysis showed an association between post-orchiectomy NLR and disease recurrence regardless of disease’s stage: HR = 1.85 (95% CI = 0.99–3.46) and HR = 1.91 (95% CI = 0.96–3.78) for stage I or stage II, respectively. A ROC curve analysis for post-orchiectomy demonstrated that NLR ≥ 2.255 was the optimal cut-off for predicting a higher probability of recurrence (AUC = 78.7% and *P* = 0.001). In the multivariable analysis, post-orchiectomy NLR ≥ 2.255 was linked to the risk of recurrence of tCa (HR = 1.51, *P* = 0.022, 95% CI = 1.108–3.677). In addition, after stratification of patients by post-orchiectomy NLR (optimal cut-off = 2.255), patients with lower NLR had significantly longer recurrence-free survival (107.7 months vs. 57.65 months, *P* < 0.001). Moreover, pre-orchiectomy NLR was higher in patients who had adjuvant chemotherapy than those without chemotherapy [2.6 (1.8–3.8) vs. 1.7 (1.3–2.5), respectively; *P* = 0.007]. Such difference was not observed post-orchiectomy [1.58 (1.05–2.13) vs. 1.82 (1.35–2.58), *P* = 0.190]. In a study by Selvi et al. [48], the data of 87 patients with TGCT were analyzed. NLR ≥ 3.23 (OR = 1.348, *P* = 0.025) increased the risk of contralateral tumor development. NLR was found to be significantly higher in the patients with bilateral disease [5.34 (4.33–7.45)] compared to patients with unilateral disease [2.76 (1.80–4.42)] (*P* = 0.001). When they divided bilateral patients into subgroups, no difference was found between synchronous-metachronous subgroups [6.13 (4.90–7.83) and 4.66 (3.62–7.45) respectively, *P* = 0.394].

Meanwhile, some other previous studies were unable to demonstrate the association between NLR and tCa survival. In the study by Arda et al. [26] on 90 tCa patients, CSS was not different between patients with NLR < 1.78 and those with NLR ≥ 1.78 (*P* = 0.378). Similarly, Yoshinaga et al. [53], in the evaluation of 63 patients with TGCT, revealed that there was no significant association between PFS and OS and NLR in multivariate analysis at the NLR cut-off of 4.1, which was the best cut off point for prediction of OS (AUC = 0.665 and *P* = 0.04). Pęksa et al. [45] reported that the best cut-off value of NLR for prediction of progression or relapse in tCa was 3.95 (AUC = 0.57). However, it was revealed that in multivariate logistic regression analysis, this value could not predict progression or relapse in either seminoma or non-seminomatous TGCT. In a study published by Bolat et al. [29], the data of 53 patients with TGCT were analyzed. Optimal threshold values of NLR were calculated as 3.55 for PFS (AUC = 0.55) and 3.0 for CSS (AUC = 0.66). However, time-to-cancer specific death between patients with an NLR of < 3.0 and those with NLR ≥ 3.0 was not different (54.72 months vs. 49.43 months, *P* = 0.119).

#### Final comment

Based on these findings, we may infer a strong link between NLR level and prognosis of tCa patients. Patients with a good prognosis defined based on a prognostic-based staging system for metastatic germ cell cancer had lower NLR compared to those with intermediate and poor prognoses. In addition, an elevated NLR predicts contralateral tumor development, lower time-to-cancer specific death, worse OS, and poorer response to chemotherapy. Due to the present controversy, debate continues about the association between NLR and CSS, PFS, and relapse-specific survival (RFS). This is an important issue for further research.

### NLR and tumor size in tCa

Six studies showed the association between NLR and tumor size in tCa [2, 34, 37, 42–44] (Table 7).

**Table 7:** General characteristics of studies on the association of NLR with tumor size in tCa

**Reference**	**Sample size**	**Study design**	**Year of publication**	**Country**	**Histo** **pathology**	**Cut off value of NLR**	**Seminomatous tCa**	**Outcome**
Hamidi et al. [34]	72	Retrospective cohort	2018	Turkey	tCa	2.39	30.50%	NLR was significantly associated with tumor size of the tCa patients.
Ilktac et al. [37]	61	Retrospective cohort	2020	Turkey	TGCT	2.56	59.00%	A significant correlation between tumor size and NLR was reported.
Olcucu et al. [44]	99	Retrospective cohort	2020	Turkey	TGCT	3.21	45.45%	NLR was significantly related to tumor size.
Kopru et al. [42]	80	Retrospective cohort	2019	Turkey	tCa	-	51.51%	No statistically significant relationship was observed between testicular tumor size and NLR.
Köşeci et al. [43]	72	Retrospective cohort	2021	Turkey	TGCT	2.5	43.00%	NLR was not significantly associated with tumor size.
Tan et al. [2]	160	Retrospective cohort	2019	Singapore	tCa	3	63.80%	No difference in the tumor size between patients with different NLR values was observed.

tCa: testicular cancer; TGCT: testicular germ cell tumor; NLR: neutrophil to lymphocyte ratio

In a study conducted by Hamidi et al. [34], the data of 72 patients with tCa were investigated, and it was reported that the tumor size of patients with NLR ≥ 2.39 was larger than those with NLR < 2.39 (3.46 ± 3.69 vs. 2.17 ± 0.9, respectively, *P* = 0.038). A significant positive correlation between tumor size and NLR was further reported in the study by Ilktac et al. [37] on 61 patients with TGCT (Pearson correlation coefficient = 0.302, *P* = 0.018). Similarly, in the study by Olcucu et al. [44] on 99 patients with TGCT, patients with NLR ≥ 3.219 had larger tumor size compared with those with lower NLR [6.3 (4–8) and 4 (2.5–6), *P* = 0.015].

On the other hand, Kopru et al. [42] observed no statistically significant relationship between testicular tumor size and NLR in a study including 66 tCa patients. In addition, no significant difference was found in tumor size between patients with NLR ≥ 2.5 and those with NLR < 2.5 (46.13 ± 17.45 and 48.49 ± 18.10, respectively, *P* = 0.6) in the study by Köşeci et al. [43] on 72 patients with TGCT.

Similarly, no difference in the tumor size between patients with NLR values of ≥ 3 and < 3 in either seminomas or non-seminomatous cancer patients (*P* = 0.594 and 0.970, respectively) was observed in the study conducted by Tan et al. [2] on 160 tCa patients.

#### Final comment

The relationship between NLR and tumor size has been subject to considerable debate in the literature. Further studies, which take this variable into account, will need to be undertaken.

### NLR and conventional tumor markers of tCa

The association between NLR and conventional tumor markers of tCa (AFP, B-HCG, and LDH) was evaluated in four studies [26, 36, 44, 46] (Table 8).

**Table 8:** General characteristics of studies on the association of NLR with conventional tumor markers of tCa

**Reference**	**Sample size**	**Study design**	**Year of publication**	**Country**	**Histo** **pathology**	**Cut off value of NLR**	**Seminomatous tCa**	**Outcome**
Arda et al. [26]	182	Retrospective cohort	2020	Turkey	TGCT	1.78	46.60%	B-HCG level was significantly higher in the patients with higher NLR.
Horsanali et al. [36]	128	Retrospective cohort	2017	Turkey	TGCT	3.72	44.80%	Levels of AFP, B-HCG, and LDH were significantly related to NLR level.
Olcucu et al. [44]	99	Retrospective cohort	2020	Turkey	TGCT	3.21	45.45%	NLR was significantly related to LDH level.
Bauzá Quetglas et al. [46]	80	Retrospective cohort	2020	Spain	tCa	2.25	62.50%	No correlation was observed between NLR and conventional tumor markers (AFP and B-HCG).

tCa: testicular cancer; TGCT: testicular germ cell tumor; NLR: neutrophil to lymphocyte ratio

In the study by Arda et al. [26] on 90 patients with tCa, pre-operative B-HCG level was higher in the patients with NLR ≥ 1.78 in comparison to those with NLR < 1.78 (5.00 ± 41.00 and 1.00 ± 4.65, respectively, *P* = 0.047). In the study by Horsanali et al. [36] on 128 tCa patients, patients with NLR ≥ 3.72 had higher levels of pre-operative AFP, B-HCG, and LDH in comparison with those with NLR < 3.72 (*P* = 0.006, < 0.001, and < 0.001, respectively). Olcucu et al. [44] categorized 99 TGCT patients into two groups of NLR < 3.219 and ≥ 3.219 and compared them in terms of conventional tumor markers (AFP, B-HCG, and LDH). Among these tumor markers, only LDH showed a statistically significant difference between two groups [223 (194.25–302.5), 337 (214–605), respectively, and *P* = 0.003]. In the study of Bauzá Quetglas et al. [46] on 80 tCa patients undergoing orchiectomy, no correlation was observed between NLR and conventional tumor markers (AFP and B-HCG) at both pre-orchiectomy (*r* value = 0.08 and 0.06, respectively) and post-orchiectomy (*r* value = 0.12 and 0.07, respectively) assessments.

#### Final comment

The inconsistency among the results of previous studies makes it impossible to judge the relationship between NLR and conventional tumor markers. Further studies on the current topic are therefore recommended.

### Dynamic change of NLR after orchiectomy

Two studies investigated the dynamic change of NLR after orchiectomy [37, 46] (Table 9).

**Table 9:** General characteristics of studies on the dynamic change of NLR after orchiectomy

**Reference**	**Sample size**	**Study design**	**Year of publication**	**Country**	**Histo** **pathology**	**Cut off value of NLR**	**Seminomatous tCa**	**Outcome**
Ilktac et al. [37]	61	Retrospective cohort	2020	Turkey	TGCT	2.56	59.00%	The NLR value of tCa patients decreased significantly after orchiectomy.
Bauzá Quetglas et al. [46]	80	Retrospective cohort	2020	Spain	tCa	2.25	62.50%	NLR was significantly reduced after orchiectomy.

tCa: testicular cancer; TGCT: testicular germ cell tumor; NLR: neutrophil to lymphocyte ratio

Ilktac et al. [37] conducted a study in which a total of 61 patients with TGCT undergoing orchiectomy were divided into two groups of localized and non-localized TGCT. They reported that NLR levels of patients with localized TGCT significantly decreased from 3.10 ± 2.13 to 1.62 ± 0.59 postoperatively (*P* = 0.010). However, the decrease was not significant in the non-localized group (*P* = 0.576). They then categorized patients into those with elevated pre-operative tumor markers (B-HCG, AFP, and LDH) and those with normal pre-operative tumor markers. The difference between pre- and post-operative NLR was significant in both groups (both *P* = 0.10). Similar results were found by Bauzá Quetglas et al. [46] that reported that NLR was significantly reduced after orchiectomy from 2.2 (1.55–3.09) to 1.77 (1.34–2.46), *P* < 0.001.

#### Final comment

Based on the findings of these investigations, we may conclude that NLR may decrease significantly after orchiectomy in tCa patients.

### Limitation

There are two major limitations in our systematic review. Due to the absence of prospective research on this topic, all of the studies included were retrospective. In order to verify these findings, further prospective research should be carried in the future. Second, we could not perform a meta-analysis due to significant heterogeneity and conflicting results in the literature. There is also a lack of a significant number of studies to be suitable for a meta-analysis.

## Discussion

Our systematic review confirms that NLR is a key diagnostic and prognostic factor in tCa. NLR was higher in tCa patients compared to healthy controls and those with benign testis pathologies, which decreased significantly after orchiectomy. An elevated NLR predicts poor prognosis, advanced stage, presence of nodal or distant metastases, contralateral tumor development, lower time-to-cancer specific death, worse OS, and poorer response to chemotherapy. However, NLR could not differentiate between seminomas and non-seminomatous tCa patients. Prospective trials are needed to establish the role of NLR in the management of tCa.

## Abbreviations

AUC: area under the curve
CSS: cancer-specific survival
EAU: European Association of Urology
HR: hazard ratio
IQR: interquartile range
LMR: lymphocyte to monocyte ratio
NLR: neutrophil to lymphocyte ratio
OR: odds ratio
OS: overall survival
PFS: progression-free survival
PLR: platelet to lymphocyte ratio
ROC: receiver operating characteristic
SD: standard deviation
SMD: standardized mean difference
tCa: testicular cancer
TGCT: testicular germ cell tumor

## Declarations

### Availability of data and materials

Datasets are available on request.

### Copyright

© The Author(s) 2024.
